# The Population-Attributable Fractions of Small-for-Gestational-Age Births: Results from the Japan Birth Cohort Consortium

**DOI:** 10.3390/nu16020186

**Published:** 2024-01-05

**Authors:** Kazue Ishitsuka, Aurélie Piedvache, Sumitaka Kobayashi, Noriyuki Iwama, Tomoko Nishimura, Masahiro Watanabe, Hirohito Metoki, Hiroyoshi Iwata, Chihiro Miyashita, Mami Ishikuro, Taku Obara, Kenichi Sakurai, Mohammad Shafiur Rahman, Keiko Tanaka, Yoshihiro Miyake, Reiko Horikawa, Reiko Kishi, Kenji J. Tsuchiya, Chisato Mori, Shinichi Kuriyama, Naho Morisaki

**Affiliations:** 1 Department of Social Medicine, National Center for Child Health and Development, 2-10-1, Okura, Setagaya-ku, Tokyo 157-8535, Japan; aurelie-p@ncchd.go.jp (A.P.); morisaki-n@ncchd.go.jp (N.M.); 2 Center for Environmental and Health Sciences, Hokkaido University, Sapporo 060-0808, Japan; sukobayashi@cehs.hokudai.ac.jp (S.K.); hiwata@cehs.hokudai.ac.jp (H.I.); miyasita@med.hokudai.ac.jp (C.M.); rkishi@med.hokudai.ac.jp (R.K.); 3 Tohoku Medical Megabank Organization, Tohoku University, 2-1 Seiryo-machi, Aoba-ku, Sendai 980-8573, Japan; noriyuki.iwama@med.tohoku.ac.jp (N.I.); m_ishikuro@med.tohoku.ac.jp (M.I.); obara-t@hosp.tohoku.ac.jp (T.O.); kuriyama@med.tohoku.ac.jp (S.K.); 4 Department of Obstetrics and Gynecology, Tohoku University Graduate School of Medicine, 1-1, Seiryomachi, Sendai 980-8574, Japan; 5 Research Center for Child Mental Development, Hamamatsu University School of Medicine, 1-20-1 Handayama, Higashi-ku, Hamamatsu 431-3192, Japan; tomoko.n@hama-med.ac.jp (T.N.); tsuchiya@hama-med.ac.jp (K.J.T.); srahman@hama-med.ac.jp (M.S.R.); 6 Department of Sustainable Health Science, Center for Preventive Medical Sciences, Chiba University, Chiba 263-8522, Japan; m_watanabe@chiba-u.jp (M.W.); cmori@faculty.chiba-u.jp (C.M.); 7 Faculty of Medicine, Tohoku Medical and Pharmaceutical University, 1-15-1, Fukumuro, Miyagino-ku, Sendai 983-8536, Japan; hmetoki@tohoku-mpu.ac.jp; 8 Department of Nutrition and Metabolic Medicine, Center for Preventive Medical Sciences, Chiba University, Chiba 263-8522, Japan; sakuraik@faculty.chiba-u.jp; 9 Department of Bioenvironmental Medicine, Graduate School of Medicine, Chiba University, Chiba 263-8522, Japan; 10 Department of Epidemiology and Preventive Medicine, Ehime University Graduate School of Medicine, 10-13 Dogo-Himata, Matsuyama 790-8577, Japan; tanaka.keiko.jn@ehime-u.ac.jp (K.T.); miyake.yoshihiro.ls@ehime-u.ac.jp (Y.M.); 11 Division of Endocrinology and Metabolism, National Center for Child Health and Development, 2-10-1 Okura, Setagaya-ku, Tokyo 157-8535, Japan; horikawa-r@ncchd.go.jp

**Keywords:** small-for-gestational-age, birth cohort, fetal growth, maternal nutrition, pregnancy

## Abstract

A fetal growth restriction is related to adverse child outcomes. We investigated risk ratios and population-attributable fractions (PAF) of small-for-gestational-age (SGA) infants in the Japanese population. Among 28,838 infants from five ongoing prospective birth cohort studies under the Japan Birth Cohort Consortium, two-stage individual-participant data meta-analyses were conducted to calculate risk ratios and PAFs for SGA in advanced maternal age, pre-pregnancy underweight, and smoking and alcohol consumption during pregnancy. Risk ratio was calculated using modified Poisson analyses with robust variance and PAF was calculated in each cohort, following common analyses protocols. Then, results from each cohort study were combined by meta-analyses using random-effects models to obtain the overall estimate for the Japanese population. In this meta-analysis, an increased risk (risk ratio, [95% confidence interval of SGA]) was significantly associated with pre-pregnancy underweight (1.72 [1.42–2.09]), gestational weight gain (1.95 [1.61–2.38]), and continued smoking during pregnancy (1.59 [1.01–2.50]). PAF of underweight, inadequate gestational weight gain, and continued smoking during pregnancy was 10.0% [4.6–15.1%], 31.4% [22.1–39.6%], and 3.2% [−4.8–10.5%], respectively. In conclusion, maternal weight status was a major contributor to SGA births in Japan. Improving maternal weight status should be prioritized to prevent fetal growth restriction.

## 1. Introduction

Globally, public health measures target the promotion of maternal and child health targets to reduce low birth weight [1]. Fetal growth restriction, a predominant cause of low birth weight, elevates the risk of not only mortality and morbidity during infancy but also poses a significant risk of developmental delay and impaired cognitive function in later childhood [2,3,4]. Furthermore, it is associated with increased risks of coronary artery diseases and diabetes in adulthood [5]. Economic estimations suggest that the high prevalence of fetal growth restriction imposes a heavy societal burden on not only population health but also the education and social service systems [6].

The identification and quantification of risk factors of fetal growth retardation or small-for-gestational-age (SGA) births are needed to prioritize preventive public health activities. Many studies have elucidated the risk factors of fetal growth restriction, and there is a general consensus that advanced maternal age, maternal smoking during pregnancy, pre-pregnancy underweight, and inadequate gestational weight gain increase the risk of fetal growth restriction or SGA births [7,8,9]. Moreover, some studies have suggested that low socio-economic status increases the risk of fetal growth restriction or SGA births [10,11]. The degree to which each risk factor contributes to the aforementioned outcomes likely varies across populations. This variation stems not only from differences in the risk each factor presents but also from their varying prevalence.

A systematic review that estimated population-attributable fractions (PAF) for risk factors of SGA in low- and middle-income countries showed that maternal undernutrition (low height, underweight, inadequate weight gain, and vitamin D deficiency) was a major contributor, which explained approximately 25% of all SGA births [12]. Studies in Western countries have shown that smoking during pregnancy had the highest PAF [13,14], and constituted 2.3–8.7% of all SGA births.

In Japan, statistics show a high level of low birthweight births even though preterm birth rates are comparable or even lower than those in other countries, suggesting that the rate of fetal growth retardation is high [15,16]. However, to our knowledge, only one study has estimated PAF for known risk factors of low birth weight, and no studies have focused on fetal growth restriction or regional differences in contributions by each factor [17].

Thus, we used a recently established birth cohort consortium in Japan to investigate the PAF for known risk factors in SGA to develop strategies for public health intervention.

## 2. Materials and Methods

### 2.1. Study Participants

We analyzed data from prospective birth cohort studies within the Japan Birth Cohort Consortium (JBiCC). The design of JBiCC has been described previously [18]. Briefly, the JBiCC was established in 2019 to investigate prenatal risk factors affecting child and maternal health. Six cohort studies participated under the JBiCC in 2019, with an additional two cohorts participating in 2022.

Eligibility criteria for the cohort study inclusion in this analysis were: (1) cohorts that had information on the birth weight of infants, gestational age at birth, infant sex, maternal smoking, and pre-pregnancy anthropometric information of mothers, and (2) cohorts that shared the results of PAF of SGA and risk ratios of SGA. Among the eight cohorts in JBiCC, three cohorts were not included in this study due to a lack of information on prepregnancy body mass index (BMI) (two cohort studies) and smoking during pregnancy (one cohort). Thus, the five studies meeting the criteria and subjected to analysis included: the Hokkaido Study on Environment and Children’s Health (Hokkaido Study), the Tohoku Medical Megabank Project Birth and Three-Generation Cohort Study (TMM BirThree Cohort Study), the Babies and their Parents’ Longitudinal Observation in the Suzuki Memorial Hospital in Intrauterine Period Study (BOSHI Study), the Chiba Study of Mother and Child Health (C-MACH Study), and the Hamamatsu Birth Cohort for Mothers and Children (HBC Study). The Hokkaido Study is a population-based prospective birth cohort study, recruiting pregnant women in the Hokkaido prefecture (*n* = 21,454). Similarly, the TMM BirThree Cohort Study is a population-based prospective birth cohort study, enrolling pregnant women in the Miyagi and Iwate prefectures (*n* = 22,493). The BOSHI Study recruited 1576 pregnant women at a maternity hospital in Sendai City, Miyagi prefecture. The C-MACH Study enrolled 433 pregnant women across three hospitals and a clinic in the Chiba and Saitama prefectures. The HBC Study involved 1138 pregnant women in a hospital and a clinic in the Shizuoka prefecture.

The participant eligibility for this study was limited to singleton and living infants. Exclusion criteria were infants with missing information on birth weight, gestational age at birth, or child sex, or those who had 42 weeks of gestation at delivery or more, wherein SGA status was incalculable due to the absence of appropriate growth standards.

Written informed consent was obtained from all participants in all cohorts participating in this study. The study protocol for this meta-analysis was approved by the Ethical Committee of the National Center of Child Health Development (approval number 2021-004, approval date 14 October 2021).

### 2.2. Outcome Variables

Anthropometric data were collected from medical records at delivery. First, SGA was defined as less than the 10th percentile of Japanese national gestational age-specific, sex-specific, and parity-specific birthweight percentiles (domestic criteria for SGA) [19]. To compare international criteria, SGA was also calculated as less than the 10th percentile of Fenton charts (international criteria for SGA) [20]. The assessment of gestational age was based on ultrasound examination and the last menstrual period.

### 2.3. Risk Factor for PAF and Covariates

Modifiable known risk factors for SGA were based on previous studies [8]: advanced maternal age, low education levels, pre-pregnancy underweight, inadequate gestational weight gain (GWG), smoking, and alcohol consumption during pregnancy.

Information on maternal pre-pregnancy height, weight, weight at delivery, and parity was obtained from the medical chart data recorded during pregnancy. BMI was classified into three categories: underweight (<18.5 kg/m^2^), normal weight (18.5–24.9 kg/m^2^), and overweight or obese (≥25 kg/m^2^) [21]. Inadequate gestational weight gain was defined as gestational weight gain, calculated as the difference between weight at delivery and before pregnancy, of <12 kg for women with a pre-pregnancy BMI < 18.5 kg/m^2^, <10 kg for women with a pre-pregnancy BMI of 18.5–24.9 kg/m^2^, and <7 kg for women with a prepregnancy BMI of 25–29.9 kg/m^2^, in accordance with Japanese guidelines [21]. As the guideline does not specify appropriate weight gain for women with a pre-pregnancy BMI >30 kg/m^2^, all women with a BMI >30 kg/m^2^ were categorized as having adequate gestational weight gain [22].

Advanced maternal age was defined as 35 years or older [8]. Maternal education level was categorized into two groups based on the reported highest academic achievement: “junior high school or high school (≤12 years)” and “junior college or four years of university or more (≥13 years)” [10,11].

Smoking and alcohol consumption during pregnancy were assessed through self-reported questionnaires administered during pregnancy. Smoking status was categorized as “never smoking”, “quit smoking after pregnancy”, and “continued smoking”. Alcohol consumption was categorized as follows: “never alcohol consumption,” “quit alcohol consumption after pregnancy awareness”, and “continued alcohol consumption after pregnancy awareness”. Table S1 shows the details of questionnaires in each cohort study.

### 2.4. Statistical Analysis

A meta-analysis of individual participant data allows more powerful and flexible analyses, better harmonization of the data, more consistent adjustment for potential confounders, and less publication bias compared with the previously performed meta-analyses of published results [23]. Thus, in this study, we conducted a two-stage meta-analysis of individual data.

First, the characteristics of study participants from each cohort study were described. The nationwide prevalence of risk factors was presented using vital statics data from the government [24,25].

Next, we calculated risk ratios (RR) and PAF of SGA in each cohort study. Unadjusted RRs for SGA and 95% confidence intervals (CI) were calculated using univariate Poisson regression with robust error variance (Model 1) [26]. Risk factors of SGA included advanced maternal age, low education levels, pre-pregnancy underweight, inadequate GWG, smoking, and alcohol consumption during pregnancy. PAF of SGA in each risk factor was also calculated in each cohort study. Subsequently, both Poisson regression and PAF were adjusted for age, parity, maternal height, smoking, alcohol consumption, and pre-pregnancy underweight (Model 2) [8,13]. For sensitivity analyses, we added other covariates available in four cohort studies, including inadequate gestational weight gain (Model 3), and maternal education (Model 4).

Third, we conducted meta-analyses of RRs and PAF using random effects in the five cohort studies. To assess heterogeneity, the I^2^ statistic was calculated. Moreover, the PAF was calculated using the prevalence of exposures based on national vital statistics of Japan and adjusted RRs from meta-analyses of all cohort studies from the consortium, using Miettinen’s formula [24,25,27]. In sensitivity analyses, we performed meta-analyses using fixed effects. Furthermore, we repeated the aforementioned analyses for international criteria for SGA.

Statistical analyses for individual cohort studies were conducted using STATA (version 14.0 or 16.0; StataCorp LP, College Station, TX, USA), SAS (version 9.4; SAS Institute Inc., Cary, NC, USA), R (version 4.1.3; R Foundation, Vienna, Austria), or SPSS version 26 (IBM Corp., Armonk, NY, USA). The PAF and its 95% CI were derived using the command punaf in STATA, the macro %NLEST in SAS, or the package pifpaf in R; these commands implement the Greenland and Drescher approach (Greenland, 1993). All meta-analyses were conducted using STATA (16 StataCorp LP, College Station, TX, USA).

## 3. Results

A total of 28,838 infants were included in this analysis. Table S1 shows the prevalence of SGA. Based on domestic criteria, the prevalence of SGA ranged from 3.5% (in the C-MACH Study) to 10.5% (in the BOSHI Study). In contrast, using international criteria, the prevalence of SGA ranged from 21.4% (in the C-MACH Study) to 28.4% (in the BOSHI Study).

Table 1 shows the prevalence of the risk factors of SGA in each cohort study and nationwide prevalence from Vital Statistics from the Japanese government. The prevalence of pre-pregnancy underweight ranged from 12.0% in the BOSHI Study to 20.8% in the HBC Study. The nationwide prevalence derived from Vital Statistics in Japan of GWG was found to be 49.6%. Notably, this prevalence varied, ranging from 45.1% in the BOSHI Study to 49.8% in the C-MACH Study. Furthermore, the nationwide prevalence of continuing smoking was established at 5.2%. This prevalence also showed variability, extending from 1.5% in the C-MACH Study to 12.0% in the Hokkaido Study. Based on nationwide prevalence derived from Vital Statistics in Japan, the prevalence of pre-pregnancy underweight, inadequate GWG, and smoking was 19.6%, 49.6%, and 5.2%.

Figures S1–S3 illustrate the outcomes of meta-analyses examining the unadjusted RR and PAF, utilizing domestic criteria for SGA in Model 1. Notably, pre-pregnancy underweight (RR 1.72, 95% CI 1.42–2.09) and inadequate GWG (RR 1.95, 95% CI 1.61–2.38) were found to be significantly associated with an elevated risk of SGA.

**Figure 1:**
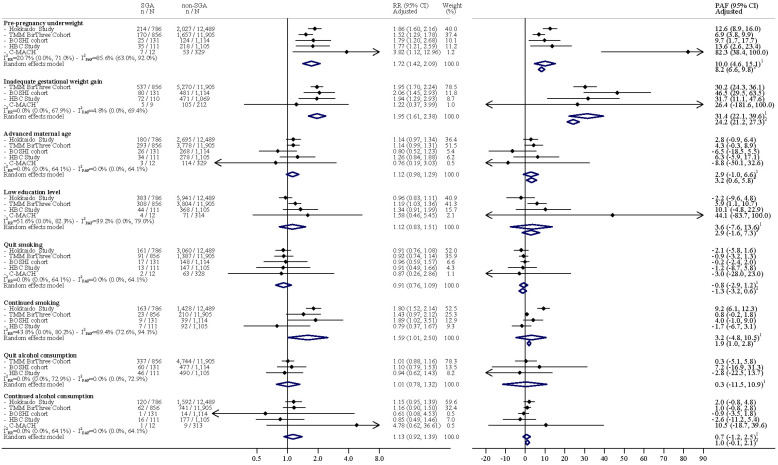
Results of meta-analyses of adjusted risk ratio and the population-attributable fraction of small-for-gestational age for pre-pregnancy underweight and inadequate gestational weight gain (Domestic criteria for small-for-gestational age). PAF: population-attributable fraction; RR: risk ratio; SGA: small-for-gestational age; CI: confidence interval; TMM BIRTHREE: The Birth and Three-Generation; HBC: Hamamatsu Birth Cohort; C-MACH: Chiba study of Mother and Children’s Health; PAF: population-attributable fraction; RR: risk ratio. I^2^_RR_, I^2^ statistics of the risk ratio; I^2^_PAF_, I^2^ statistics of the population-attributable fraction. ^1^ PAF was calculated using PAF derived from each cohort study. ^2^ PAF was calculated using the pooled risk ratio estimated and the nationwide prevalence.

Figure 1 illustrates the outcomes of meta-analyses on adjusted RR and PAF, adhering to domestic criteria in Model 2. Specifically, pre-pregnancy underweight (RR 1.72, 95% CI 1.42–2.09), inadequate GWG (RR 1.95, 95% CI 1.61–2.38), and continuous smoking during pregnancy (RR 1.59, 95% CI 1.01–2.50) were each significantly associated with a heightened risk of SGA births.

The highest PAF was seen for inadequate GWG (31.4% [95% CI 22.1–39.6%]), followed by pre-pregnancy underweight (10.0% [95% CI 4.6–15.1%]). The PAF of continued smoking in pregnancy was 3.2% (95% CI −4.8–10.5%).

Furthermore, the PAF calculated based on nationwide prevalence data of risk factors from Vital Statistics by the government, as opposed to the PAF derived from meta-analyses of individual cohort studies, yielded similar results. The most significant PAF was noted for inadequate GWG, representing 24.2% ([95%CI 21.2–27.3%]), succeeded by pre-pregnancy underweight at 8.2% ([95%CI 6.6–9.8%]). Moreover, the PAF attributable to persistent smoking was 1.9% ([95%CI 1.0–2.8%]).

In our meta-analyses of cohort studies, there was no association between advanced maternal age (1.12 [95%CI 0.98–1.29]) and SGA. This lack of association was also observed in the analysis of individual cohort studies. Similarly, our meta-analyses revealed no association between low education level (1.12 [95%CI 0.83–1.51]) and SGA. However, in the analysis of individual cohort studies, a low education level was associated with an increased risk of SGA in the TMM BirThree Cohort Study (1.19 [95%CI 1.03–1.36]).

The highest heterogeneity of RR was observed for maternal education (51.6% [95% CI 0.0–82.3%]). In contrast, the lowest RR heterogeneity was noted in cases of advanced maternal age, inadequate GWG, pre-pregnancy underweight, cessation of smoking, and discontinued, as well as continued, alcohol consumption, all registering 0.0% [95% CI 0.0–72.9%]. Conversely, the highest PAF heterogeneity was recorded in continued smoking (89.4% [95% CI 72.6–94.1%]), while the lowest was found in advanced maternal age, smoking cessation, and ongoing alcohol consumption, each with 0.0% [95% CI 0.0–64.1%].

Adding education levels (Model 3) and inadequate GWG (Model 4) as covariates to regression models (Model 2) showed similar results (Figure S4). Furthermore, analyses using International Criteria for SGA produced similar results (Figures S5–S11).

Finally, sensitivity analyses employing meta-analyses of fixed effect models produced similar results (Table S3).

## 4. Discussion

In this two-stage individual meta-analysis, we found that maternal pre-pregnancy underweight, inadequate gestational weight gain, and continued smoking during pregnancy were significantly associated with increased risk of SGA in infants. Inadequate GWG and pre-pregnancy underweight were major contributors to SGA in the Japanese population.

Our meta-analysis found that inadequate gestational weight gain was the most significant contributory factor of SGA in all cohorts, with one-third of all cases explainable by this characteristic. Furthermore, PAF was high (26–46%) in all cohorts. Though few studies have reported the estimated contribution of inadequate GWG on SGA, this estimate is much higher compared with that reported in Canada (9.2%), possibly due to the higher prevalence in our study (our study 50%, Canada 18.6%) as the increase in risk (our study RR 1.95, Canada OR 1.56) was similar in both populations [14]. The PAF of SGA due to inadequate GWG is higher in this study compared with the results from a systematic review of PAF of SGA in low- and middle-income countries (2.5%) [12]. Our findings align with those from a Japanese cohort study, which indicated that inadequate GWG presents the highest PAF among risk factors for low birth weight (16.5%) [17]. These outcomes reinforce the notion that inadequate GWG is a significant contributor to fetal growth restriction in Japan. This observation suggests that optimizing gestational weight gain could be a promising intervention, regardless of the region. Notably, a recent development in Japan is a GWG chart based on gestational age [30]. This tool aids gynecologists and dieticians in providing tailored weight guidance and nutritional advice specific to each stage of a woman’s pregnancy. Further research is imperative to identify nutrient deficiencies in women experiencing inadequate GWG and to determine optimal nutritional recommendations for achieving sufficient GWG.

In our meta-analysis, the second largest contributor was pre-pregnancy underweight, which explained one in ten cases. Compared with that of a previous study from Canada, the PAF was slightly higher (our study 9.4%, Canada 5.3%), with higher prevalence (our study 20%, Canada 6%) and similar risk (our study: RR 1.73, Canada: odds ratio 2.47). This may be explained by the difference in the prevalence of underweight. Though overweight and obesity have been recognized as a major public health problem in most high-income countries, the double burden of malnutrition, characterized as the co-existence of underweight and overweight individuals, is an emerging problem in many Asian countries [28]. The prevalence of underweight is approximately 5% in North America and Europe, whereas Japan has the seventh highest prevalence among all countries [29], with some studies indicating a prevalence of 16–18% [17,31]. Our study suggests that for Japan, optimizing pre-pregnancy weight during the preconception period is a promising intervention to reduce SGA, similar to strategies that have been suggested in low- and middle-income countries with a high prevalence of maternal undernutrition. While the causes of being underweight vary, unnecessary weight loss practices are prevalent in Japan [32]. Thinness is often considered the epitome of beauty [33,34]. Importantly, a study indicates that Japanese women tend to favor images of women with a lower BMI compared with the preferences of women in the UK [35]. Therefore, strategies for the prevention and intervention of underweight during the preconception period must encompass addressing inappropriate intended weight loss.

Previous studies conducted in Australia have shown that smoking is a significant contributor to the PAF of SGA births [13]. Our meta-analyses demonstrated that the PAF of SGA associated with maternal smoking during pregnancy is approximately 1.9%. Although this percentage might seem relatively low, it is important to note that the variability in PAF for smoking across different cohorts was more pronounced compared with the RR, where variations could be largely attributed to disparities in measurement methodologies. This variation may reflect differences in the prevalence of continued smoking. In this study, a high prevalence of smoking (12%) and a notable PAF (9.7%) were observed in Hokkaido, in contrast to Tohoku, where both the smoking prevalence and PAF were markedly lower (1.8%). Regions with elevated smoking rates demonstrated a significant contribution of smoking to SGA. Consequently, in these areas, a reduction in smoking rates might result in a decrease in the incidence of SGA infants.

Education is one of the most extensively studied social determinants of health. Low education can pose barriers to access to information and knowledge on maternal and child health and reduce social networks to enhance fetal growth. Studies have indicated that low education increases the risk of SGA in North America, Australia, New Zealand, and Europe [10,11,36,37,38,39]. However, these associations were not observed in Italy, Portugal, Spain, and Ireland [40]. Our meta-analysis showed that maternal education levels were not associated with an increased risk of SGA in Japan. In the analysis of individual cohorts, a single cohort study demonstrated that lower maternal education elevates the risk of SGA births. This observed variation could be attributed to regional disparities in the impact of inequality. Additional research is required to substantiate this association.

The age of childbearing has been delayed in high-income countries [41]. Our cohort studies revealed that the prevalence of advanced maternal age ranged from 21.7 to 34.3%. Importantly, as women age, there is a decrease in uteroplacental perfusion, leading to fetal growth restriction [42]. This decrease in uteroplacental perfusion occurs as women grow older, independently of parity, and impacts fetal growth [42]. Notably, increasing age is associated with heightened concerns about the risk of intrauterine growth restriction, attributed to a rise in chronic diseases and intrapartum complications, thereby elevating the risk of SGA infants. A previous international meta-analysis study has indicated that advanced maternal age is a risk factor for SGA [43]. However, our study found no association between advanced maternal age and SGA in the unadjusted model. Specifically, advanced maternal age may offer certain protective factors against SGA, including psychological and social advantages [44]. These factors could potentially offset the adverse effects of advancing age. Furthermore, even after adjusting for socio-economic factors, pre-pregnancy BMI, and smoking habits, our study continued to observe no significant associations between advanced maternal age and SGA.

Globally, approximately one-third of women of reproductive age consume alcohol [45]. In our cohort studies, over 40% of women reported alcohol consumption prior to pregnancy. A majority of pregnant women stop alcohol use upon becoming aware of their pregnancy; however, some of them persist in consuming alcohol. A systematic review has established that prenatal alcohol exposure heightens the risk of fetal growth restriction [46]. Additionally, existing studies have suggested that alcohol intake during the preconception period may adversely affect fetal growth [47,48]. Importantly, prenatal alcohol exposure can alter placental function and trigger sustained stress responses in the placenta [49]. This exposure may also lead to epigenetic modifications that impact embryonic and placental development [50]. In our recent meta-analysis, neither cessation of alcohol consumption after pregnancy recognition nor ongoing alcohol use was linked to a significantly increased risk of SGA infants. While numerous studies have suggested that prenatal alcohol exposure raises the risk of fetal growth restriction regardless of the quantity of alcohol consumed, some other studies do not show an elevated risk of SGA associated with low-level alcohol consumption [46,51]. Consequently, further research is imperative to ascertain the specific alcohol consumption patterns that increase the risk of SGA.

International growth curves are designed for global applicability, encompassing a diverse array of countries and ethnic groups [20]. However, these curves may not fully reflect the distinct characteristics of specific regions, as their development is intended for broad use. Variations in growth curves between countries can be attributed to differences in maternal characteristics. For example, the average birth weight in Japan is generally lower than that in Western countries, which could be a result of the lower pre-pregnancy BMI observed in Japanese women [20]. To facilitate more effective international comparisons in the future, we explored the PAF using two different criteria sets: one employing domestic growth curves and the other, international growth curves. Our results revealed that the PAF for SGA when based on international criteria, yielded similar outcomes.

The present study possesses several significant strengths. Initially, meta-analyses were performed subsequent to the development of a consensus protocol. This protocol enabled the standardization of statistical methods across our cohort studies. Second, while systematic reviews that rely on published papers might be subject to publication bias—where studies with positive results are more likely to be published [52]—meta-analyses from the Birth Cohort Consortium mitigate this issue by utilizing all available data. Finally, the large sample sizes drawn from various regions enhance the broad applicability of our findings. Nevertheless, certain limitations must be acknowledged. The data on pre-pregnancy weight status, as reported by women, might have led to an underestimation or overestimation of their weight. However, the categorization of self-reported pre-pregnancy weight status has been validated as reliable [53]. The observed prevalence of underweight was in line with that from prior studies conducted in Japan [17,31]. Additionally, the self-reporting of smoking status may have led to an underestimation of its prevalence. The potential for underreporting both smoking and alcohol consumption consistently presents a concern in epidemiological research.

## 5. Conclusions

In conclusion, our results showed that pre-pregnancy underweight, smoking, and inadequate GWG were associated with an increased risk of SGA. Our results also showed that maternal nutritional status was a major contributor to PAF in SGA in the Japanese population. A reduction in inadequate weight gain and pre-pregnancy underweight are aspects that can be prioritized in public health interventions to reduce SGA in Japan.

## Figures and Tables

**Table 1:** Prevalence of risk factors for small-for-gestational age in each cohort study (%).

		Hokkaido Study	TMM BIRTHREE Cohort Study	HBC Study	BOSHI Study	C-MACH Study	Nationwide
Age	<35 years old	78.3	68.1	74.3	76.4	65.7	70.9
	≥35 years old	21.7	31.9	25.7	23.6	34.3	29.1
Parity	Nulliparous	41.3	45.7	49.7	42.7	36.1	41.5
	Multiparous	58.7	54.3	50.3	57.3	63.9	58.5
Education	High school	47.6	32.2	33.9	NA	23.0	27.0
	College and more	52.4	67.8	66.1	NA	77.0	73.0
Pre-pregnancy BMI	<18.5 kg/m^2^	16.9	14.3	20.8	12.0	17.6	19.6
	≥18.5 kg/m^2^	83.1	85.7	79.2	88.0	82.4	80.4
GWG	Adequate	NA	54.5	53.9	54.9	50.2	50.4
	Inadequate	NA	45.5	46.1	45.1	49.8	49.6
Smoking	Never	63.8	86.6	78.7	82.9	79.4	81.1
	Quit smoking after pregnancy	24.3	11.6	13.2	13.3	19.1	13.7
	Continued smoking	12.0	1.8	8.1	3.9	1.5	5.2
Alcohol consumption	Never	87.1	53.9	40.0	55.7	96.9	91.3
	Quit alcohol consumption	NA	39.8	44.1	43.1	NA	NA
	Continued alcohol consumption	12.9	6.3	15.9	1.2	3.1	8.7

BMI: body mass index; GWG: gestational weight gain; NA: not available; TMM BIRTHREE: The Birth and Three-Generation; HBC: Hamamatsu Birth Cohort; C-MACH: Chiba study of Mother and Children’s Health. Hokkaido Study did not collect information on GWG. BOSHI Study did not collect information on maternal education. Hokkaido and C-MACH did not collect information on alcohol after pregnancy awareness. Prevalence of risk factors nationwide was assessed through nationwide surveys using vital statics data from the government [28,29].

## Data Availability

Aggregated data used in this study are available upon request.

## Supplementary Materials

The following supporting information can be downloaded at: https://www.mdpi.com/article/10.3390/nu16020186/s1, Table S1: Questionnaires to assess smoking and alcohol consumption; Table S2: Prevalence of small-for-gestational age in five cohort studies; Table S3: Sensitivity analyses of the meta-analyses using the fixed-effect and random-effect models with pre-set heterogeneity (Domestic criteria for small-for-gestational age); Figure S1: Results of meta-analyses of unadjusted risk ratio and population-attributable fraction of small-for-gestational age for pre-pregnancy underweight and inadequate gestational weight gain (Domestic criteria for small-for-gestational age); Figure S2: Results of meta-analyses of unadjusted risk ratio and population-attributable fraction of small-for-gestational age for advanced maternal age and low educational level (Domestic criteria for small-for-gestational age); Figure S3: Results of meta-analyses of unadjusted risk ratio and population-attributable fraction of small-for-gestational age for smoking and alcohol consumption (Domestic criteria for small-for-gestational age); Figure S4: Results of the risk ratio and population-attributable fraction in models 3 and 4 (Domestic criteria for small-for-gestational age); Figure S5: Results of the meta-analyses of the unadjusted risk ratio and population-attributable fraction of small-for-gestational age for pre-pregnancy underweight and inadequate gestational weight gain (International criteria for small-for-gestational age); Figure S6: Results of the meta-analyses of the unadjusted risk ratio and population-attributable fraction of small-for-gestational age for advanced maternal age, parity, and low educational level (International criteria for small-for-gestational age); Figure S7: Results of the meta-analyses of the unadjusted risk ratio and population-attributable fraction of small-for-gestational age for smoking and alcohol consumption (International criteria for small-for-gestational age); Figure S8: Results of the meta-analyses of the adjusted risk ratio and population-attributable fraction of small-for-gestational age for pre-pregnancy underweight and inadequate gestational weight gain (International criteria for small-for-gestational age); Figure S9: Results of the meta-analyses of the adjusted risk ratio and population-attributable fraction of small-for-gestational age for advanced maternal age, parity, and low educational level (International criteria for small-for-gestational age); Figure S10: Results of the meta-analyses of the adjusted risk ratio and population-attributable fraction of small-for-gestational age for smoking and alcohol consumption (International criteria for small-for-gestational age); Figure S11: Results of the risk ratio and population-attributable fraction in models 3 and 4 (International criteria for small-for-gestational age).

## References

[1] World Health Organization (2014). Global Nutrition Targets 2025: Low Birth Weight Policy Brief: WHO, 2014. https://www.who.int/publications/i/item/WHO-NMH-NHD-14.5.

[2] Levine T.A., Grunau R.E., McAuliffe F.M., Pinnamaneni R., Foran A., Alderdice F.A. (2015). Early childhood neurodevelopment after intrauterine growth restriction: A systematic review. Pediatrics.

[3] Murray E., Fernandes M., Fazel M., Kennedy S.H., Villar J., Stein A. (2015). Differential effect of intrauterine growth restriction on childhood neurodevelopment: A systematic review. BJOG Int. J. Obstet. Gynaecol..

[4] Longo S., Bollani L., Decembrino L., Di Comite A., Angelini M., Stronati M. (2013). Short-term and long-term sequelae in intrauterine growth retardation (IUGR). J. Matern. Fetal Neonatal Med..

[5] Chen J., Chen P., Bo T., Luo K. (2016). Cognitive and Behavioral Outcomes of Intrauterine Growth Restriction School-Age Children. Pediatrics.

[6] Barker D.J. (1997). Maternal nutrition, fetal nutrition, and disease in later life. Nutrition.

[7] Petrou S., Sach T., Davidson L. (2001). The long-term costs of preterm birth and low birth weight: Results of a systematic review. Child Care Health Dev..

[8] McCowan L., Horgan R.P. (2009). Risk factors for small for gestational age infants. Best Pract. Res. Clin. Obstet. Gynaecol..

[9] Goldstein R.F., Abell S.K., Ranasinha S., Misso M., Boyle J.A., Black M.H., Li N., Hu G., Corrado F., Rode L. (2017). Association of Gestational Weight Gain With Maternal and Infant Outcomes: A Systematic Review and Meta-analysis. JAMA.

[10] Blumenshine P., Egerter S., Barclay C.J., Cubbin C., Braveman P.A. (2010). Socioeconomic disparities in adverse birth outcomes: A systematic review. Am. J. Prev. Med..

[11] Ruiz M., Goldblatt P., Morrison J., Kukla L., Švancara J., Riitta-Järvelin M., Taanila A., Saurel-Cubizolles M.J., Lioret S., Bakoula C. (2015). Mother’s education and the risk of preterm and small for gestational age birth: A DRIVERS meta-analysis of 12 European cohorts. J. Epidemiol. Community Health.

[12] Gurung S., Tong H.H., Bryce E., Katz J., Lee A.C., Black R.E., Walker N. (2022). A systematic review on estimating population attributable fraction for risk factors for small-for-gestational-age births in 81 low- and middle-income countries. J. Glob. Health.

[13] Taylor L.K., Lee Y.Y., Lim K., Simpson J.M., Roberts C.L., Morris J. (2013). Potential prevention of small for gestational age in Australia: A population-based linkage study. BMC Pregnancy Childbirth.

[14] Dzakpasu S., Fahey J., Kirby R.S., Tough S.C., Chalmers B., Heaman M.I., Bartholomew S., Biringer A., Darling E.K., Lee L.S. (2015). Contribution of prepregnancy body mass index and gestational weight gain to adverse neonatal outcomes: Population attributable fractions for Canada. BMC Pregnancy Childbirth.

[15] Chawanpaiboon S., Vogel J.P., Moller A.B., Lumbiganon P., Petzold M., Hogan D., Landoulsi S., Jampathong N., Kongwattanakul K., Laopaiboon M. (2019). Global, regional, and national estimates of levels of preterm birth in 2014: A systematic review and modelling analysis. Lancet Glob. Health.

[16] OECD (2020). Low Birth Weight in: OECD Family Database.

[17] Nishihama Y., Nakayama S.F., Tabuchi T. (2022). Population attributable fraction of risk factors for low birth weight in the Japan Environment and Children’s Study. Environ. Int..

[18] Morisaki N., Obara T., Piedvache A., Kobayashi S., Miyashita C., Nishimura T., Ishikuro M., Sata F., Horikawa R., Mori C. (2022). Association between smoking and hypertension in pregnancy among Japanese women: A meta-analysis of birth cohort studies in the Japan Birth Cohort Consortium (JBiCC) and JECS. J. Epidemiol..

[19] Itabashi K., Miura F., Uehara R., Nakamura Y. (2014). New Japanese neonatal anthropometric charts for gestational age at birth. Pediatr. Int..

[20] Kiserud T., Benachi A., Hecher K., Perez R.G., Carvalho J., Piaggio G., Platt L.D. (2018). The World Health Organization fetal growth charts: Concept, findings, interpretation, and application. Am. J. Obs. Gynecol..

[21] Japan Society of Obstetrics and Gynecology (2021). Guideline for gestational weight gain. Acta Obstet. Gynaecol. Jpn..

[22] Chen A., Xu F., Xie C., Wu T., Vuong A.M., Miao M., Yuan W., DeFranco E.A. (2015). Gestational Weight Gain Trend and Population Attributable Risks of Adverse Fetal Growth Outcomes in Ohio. Paediatr. Perinat Epidemiol..

[23] Santos S., Voerman E., Amiano P., Barros H., Beilin L.J., Bergström A., Charles M.A., Chatzi L., Chevrier C., Chrousos G.P. (2019). Impact of maternal body mass index and gestational weight gain on pregnancy complications: An individual participant data meta-analysis of European, North American and Australian cohorts. BJOG Int. J. Obstet. Gynaecol..

[24] Ministry of Health, Labour and Welfare (2019). Vital Statistics. http://www.mhlw.go.jp/toukei/saikin/hw/jinkou/geppo/nengai11/kekka02.html.

[25] Ministry of Health, Labour and Welfare (2010). National Nutrition Survey on Preschool Children. https://www.mhlw.go.jp/toukei/list/73-22a.html#mokuteki.

[26] Zou G. (2004). A modified poisson regression approach to prospective studies with binary data. Am. J. Epidemiol..

[27] Mansournia M.A., Altman D.G. (2018). Population attributable fraction. BMJ.

[28] Biswas T., Magalhaes R.J.S., Townsend N., Das S.K., Mamun A. (2020). Double Burden of Underweight and Overweight among Women in South and Southeast Asia: A Systematic Review and Meta-analysis. Adv. Nutr..

[29] NCD Risk Factor Collaboration (NCD-RisC) (2016). Trends in adult body-mass index in 200 countries from 1975 to 2014: A pooled analysis of 1698 population-based measurement studies with 19.2 million participants. Lancet.

[30] Morisaki N., Piedvache A., Morokuma S., Nakahara K., Ogawa M., Kato K., Sanefuji M., Shibata E., Tsuji M., Shimono M. (2023). Gestational Weight Gain Growth Charts Adapted to Japanese Pregnancies Using a Bayesian Approach in a Longitudinal Study: The Japan Environment and Children’s Study. J. Epidemiol..

[31] Enomoto K., Aoki S., Toma R., Fujiwara K., Sakamaki K., Hirahara F. (2016). Pregnancy Outcomes Based on Pre-Pregnancy Body Mass Index in Japanese Women. PLoS ONE.

[32] Hayashi F., Takimoto H., Yoshita K., Yoshiike N. (2006). Perceived body size and desire for thinness of young Japanese women: A population-based survey. Br. J. Nutr..

[33] Owen P.R., Laurel-Seller E. (2000). Weight and Shape Ideals: Thin Is Dangerously In. J. Appl. Soc. Psychol..

[34] Swami V., Frederick D.A., Aavik T., Alcalay L., Allik J., Anderson D., Andrianto S., Arora A., Brännström A., Cunningham J. (2010). The attractive female body weight and female body dissatisfaction in 26 countries across 10 world regions: Results of the international body project I. Pers. Soc. Psychol. Bull..

[35] Swami V., Caprario C., Tovée M.J., Furnham A. (2006). Female physical attractiveness in Britain and Japan: A cross-cultural study. Eur. J. Personal..

[36] Parker J.D., Schoendorf K.C., Kiely J.L. (1994). Associations between measures of socioeconomic status and low birth weight, small for gestational age, and premature delivery in the United States. Ann. Epidemiol..

[37] Bushnik T., Yang S., Kaufman J.S., Kramer M.S., Wilkins R. (2017). Socioeconomic disparities in small-for-gestational-age birth and preterm birth. Health Rep..

[38] Thompson J.M., Clark P.M., Robinson E., Becroft D.M., Pattison N.S., Glavish N., Pryor J.E., Wild C.J., Rees K., Mitchell E.A. (2001). Risk factors for small-for-gestational-age babies: The Auckland Birthweight Collaborative Study. J. Paediatr. Child Health.

[39] Beard J.R., Lincoln D., Donoghue D., Taylor D., Summerhayes R., Dunn T.M., Earnest A., Morgan G. (2009). Socioeconomic and maternal determinants of small-for-gestational age births: Patterns of increasing disparity. Acta Obs. Gynecol. Scand..

[40] Pillas D., Marmot M., Naicker K., Goldblatt P., Morrison J., Pikhart H. (2014). Social inequalities in early childhood health and development: A European-wide systematic review. Pediatr. Res..

[41] United Nation (2021). Mean Age of Women at Birth of First Child. https://w3.unece.org/PXWeb/en/Table?IndicatorCode=34.

[42] Lean S.C., Heazell A.E.P., Dilworth M.R., Mills T.A., Jones R.L. (2017). Placental Dysfunction Underlies Increased Risk of Fetal Growth Restriction and Stillbirth in Advanced Maternal Age Women. Sci. Rep..

[43] Lean S.C., Derricott H., Jones R.L., Heazell A.E.P. (2017). Advanced maternal age and adverse pregnancy outcomes: A systematic review and meta-analysis. PLoS ONE.

[44] Newburn-Cook C.V., Onyskiw J.E. (2005). Is older maternal age a risk factor for preterm birth and fetal growth restriction? A systematic review. Health Care Women Int..

[45] World Health Organization (2018). Global Status Report on Alcohol and Health.

[46] Mamluk L., Edwards H.B., Savović J., Leach V., Jones T., Moore T.H.M., Ijaz S., Lewis S.J., Donovan J.L., Lawlor D. (2017). Low alcohol consumption and pregnancy and childhood outcomes: Time to change guidelines indicating apparently ‘safe’ levels of alcohol during pregnancy? A systematic review and meta-analyses. BMJ Open.

[47] Nykjaer C., Alwan N.A., Greenwood D.C., Simpson N.A., Hay A.W., White K.L., Cade J.E. (2014). Maternal alcohol intake prior to and during pregnancy and risk of adverse birth outcomes: Evidence from a British cohort. J. Epidemiol. Community Health.

[48] Lundsberg L.S., Illuzzi J.L., Belanger K., Triche E.W., Bracken M.B. (2015). Low-to-moderate prenatal alcohol consumption and the risk of selected birth outcomes: A prospective cohort study. Ann. Epidemiol..

[49] Gardebjer E.M., Cuffe J.S., Pantaleon M., Wlodek M.E., Moritz K.M. (2014). Periconceptional alcohol consumption causes fetal growth restriction and increases glycogen accumulation in the late gestation rat placenta. Placenta.

[50] Pielage M., El Marroun H., Odendaal H.J., Willemsen S.P., Hillegers M.H.J., Steegers E.A.P., Rousian M. (2023). Alcohol exposure before and during pregnancy is associated with reduced fetal growth: The Safe Passage Study. BMC Med..

[51] Patra J., Bakker R., Irving H., Jaddoe V.W., Malini S., Rehm J. (2011). Dose-response relationship between alcohol consumption before and during pregnancy and the risks of low birthweight, preterm birth and small for gestational age (SGA)-a systematic review and meta-analyses. BJOG Int. J. Obstet. Gynaecol..

[52] Kicinski M., Springate D.A., Kontopantelis E. (2015). Publication bias in meta-analyses from the Cochrane Database of Systematic Reviews. Stat. Med..

[53] Shin D., Chung H., Weatherspoon L., Song W.O. (2014). Validity of prepregnancy weight status estimated from self-reported height and weight. Matern. Child Health J..

