# Supplementary material for: The Population-Attributable Fractions of Small-for-Gestational-Age Births: Results from the Japan Birth Cohort Consortium
Source: Nutrients. 2024 Jan 5;16(2):186. doi: 10.3390/nu16020186 (PMC10820645; doi:10.3390/nu16020186)
Supplement: Supplementary file 1 [file nutrients-16-00186-s001.zip › nutrients-2734982-supplementary.pdf]

List of Tables

Table S1. Questionnaires to assess smoking and alcohol consumption.

Table S2. Prevalence of small-for-gestational age in five cohort studies

Table S3. Sensitivity analyses of the meta-analyses using the fixed-effect and random-effect models with pre-set heterogeneity (Domestic criteria for small-for-gestational age).

List of Figures

Figure S1 Results of meta-analyses of unadjusted risk ratio and population-attributable fraction of small-for-gestational age for pre-pregnancy underweight and inadequate gestational weight gain (Domestic criteria for small-for-gestational age).

Figure S2 Results of meta-analyses of unadjusted risk ratio and population-attributable fraction of small-for-gestational age for advanced maternal age and low educational level (Domestic criteria for small-for-gestational age).

Figure S3 Results of meta-analyses of unadjusted risk ratio and population-attributable fraction of small-for-gestational age for smoking and alcohol consumption (Domestic criteria for small-for-gestational age).

Figure S4 Results of the risk ratio and population-attributable fraction in models 3 and 4 (Domestic criteria for small-for- gestational age).

Figure S5 Results of the meta-analyses of the unadjusted risk ratio and population-attributable fraction of small-for-gestational for age for pre-pregnancy underweight and inadequate gestational weight gain (International criteria for small-for-gestational age).

Figure S6 Results of the meta-analyses of the unadjusted risk ratio and population-attributable fraction of small-for-gestational age for advanced maternal age and low educational level (International criteria for small-for-gestational age).

Figure S7 Results of the meta-analyses of the unadjusted risk ratio and population-attributable fraction of small-for-gestational age for smoking and alcohol consumption (International criteria for small-for-gestational age).

Figure S8 Results of the meta-analyses of the adjusted risk ratio and population-attributable fraction of small-for-gestational age for pre-pregnancy underweight and inadequate gestational weight gain (International criteria for small-for-gestational age).

Figure S9 Results of the meta-analyses of the adjusted risk ratio and population-attributable fraction of small-for-gestational age for advanced maternal age and low educational level (International criteria for small-for-gestational age).

Figure S10 Results of the meta-analyses of the adjusted risk ratio and population-attributable fraction of small-for-gestational age for smoking and alcohol consumption (International criteria for small-for-gestational age).

Figure S11 Results of the risk ratio and population-attributable fraction in models 3 and 4 (International criteria for small-for- gestational age).

Table S1. Questionnaires to assess smoking and alcohol consumption.

	Questions asked in study	
	Smoking	Alcohol consumption
Hokkaido Study	Never	Never
	Ever Smoked but did not smoke before pregnancy	Drinking weekly
	Ever Smoked but smoked before pregnancy	Drinking monthly
	Smoked before pregnancy but quit during pregnancy (12 weeks or after)	Drinking daily
	Smoked before pregnancy and continued during pregnancy (12 weeks or after)	
TMM BirThree Cohort Study	Never	Never
	Used to smoke but quit before getting pregnant	Rarely drink
	Used to smoke but quit after getting pregnant (before 12 weeks)	Quit
	Used to smoke but quit after getting pregnant (12 weeks or after)	Drink
	I smoke	
	no answer	
HBC Study	Never smoked.	Never
	Never smoked after pregnancy.	Never drinking after pregnancy.
	Never smoked after 12 weeks of pregnancy.	Never drinking after 12 weeks of pregnancy.
	Never smoked after 24 weeks of pregnancy.	Never drinking after 24 weeks of pregnancy.
	Little or no smoking after 24 weeks of pregnancy.	Little or no drinking after 24 weeks of pregnancy.
	Smoke every day.	Drinking every day.
	no answer	no answer
BOSHI cohort Study	Do not smoke	Never
	Smoker and number of cigarettes per day before pregnancy is 0 or missing, and number of cigarettes per day is 0 or missing	Drink before pregnancy
	Smoker and number of cigarettes per day before pregnancy is 1 or above, and number of cigarettes per day is 0 or missing	Currently drinking
	Smoker and number of cigarettes per day before pregnancy is 0 or missing, and number of cigarettes per day is 1 or above	
	Smoker and number of cigarettes per day before pregnancy is 1 or above, and number of cigarettes per day is 1 or above	
C-MACH Study	Never	Never
	Quit smoking	Once a month
	Smoking	Every two weeks
		Once a week

	2-3 times per week
	Every day

Table S2. Prevalence of small-for-gestational age in five cohort studies.

		Hokkaido Study (N=13,275)	TMM BirThree Cohort Study (N=12,761)	HBC Study (N=1,216)	BOSHI cohort Study (N=1,245)	C-MACH Study (N=341)
Domestic criteria for small-for-gestational age	Japanese curves, birth weight <10th percentile	786 (5.9%)	856 (6.7%)	111 (9.1%)	131 (10.5%)	12 (3.5%)
International criteria for small-for-gestational age	Fenton curves, birth weight <10th percentile	3,120 (23.5%)	3,072 (24.0%)	288 (23.7%)	353 (28.4%)	73 (21.4%)

Table S3. Sensitivity analyses of the meta-analyses using the fixed-effect and random-effect models with pre-set heterogeneity (Domestic criteria for small-for-gestational age).

			Model 1	Model 2	Model 3	Model 4
**Pre-pregnancy underweight**	Fixed effect model	RR	1.69 (1.52, 1.87)	1.71 (1.54, 1.89)	1.35 (1.18, 1.56)	1.33 (1.15, 1.54)
		PAF[1]	9.0 (6.8, 11.1)	9.4 (7.2, 11.5)	4.6 (2.0, 7.1)	4.2 (1.5, 6.9)
		PAF[2]	8.0 (6.8, 9.2)	8.1 (7.0, 9.3)	5.1 (3.1, 7.1)	4.9 (2.7, 7.1)
	Random effect model	RR	1.76 (1.32, 2.34)	1.79 (1.34, 2.38)	1.55 (0.98, 2.46)	1.60 (0.91, 2.81)
		PAF[1]	10.1 (3.4, 16.3)	10.4 (3.7, 16.7)	6.7 (-4.5, 16.7)	6.9 (-12.7, 23.1)
		PAF[2]	8.4 (5.3, 11.6)	8.6 (5.5, 11.8)	7.0 (1.2, 12.8)	7.3 (0.4, 14.3)
**Inadequate gestational weight gain**	Fixed effect model	RR	2.01 (1.79, 2.26)	1.95 (1.73, 2.20)	Not applicable	1.96 (1.73, 2.23)
		PAF[1]	32.8 (27.4, 37.8)	31.4 (25.8, 36.6)	Not applicable	30.8 (25.0, 36.2)
		PAF[2]	25.0 (22.0, 27.9)	24.2 (21.2, 27.3)	Not applicable	24.3 (21.1, 27.6)
	Random effect model	RR	1.96 (1.31, 2.95)	1.90 (1.25, 2.88)	Not applicable	1.82 (1.03, 3.22)
		PAF[1]	36.4 (12.1, 54.0)	35.8 (10.1, 54.2)	Not applicable	31.2 (-3.9, 54.4)
		PAF[2]	24.4 (14.1, 34.6)	23.4 (12.5, 34.4)	Not applicable	22.4 (6.9, 37.9)
**Maternal older age**	Fixed effect model	RR	1.08 (0.98, 1.19)	1.12 (1.02, 1.24)	1.06 (0.94, 1.20)	1.12 (0.98, 1.27)
		PAF[1]	1.9 (-0.8, 4.5)	2.9 (0.1, 5.6)	1.2 (-2.8, 5.1)	3.5 (-0.8, 7.7)
		PAF[2]	2.2 (-0.4, 4.9)	3.2 (0.6, 5.8)	1.6 (-1.8, 5.0)	3.1 (-0.3, 6.5)
	Random effect model	RR	1.04 (0.78, 1.38)	1.07 (0.80, 1.43)	0.95 (0.60, 1.49)	1.08 (0.61, 1.91)
		PAF[1]	0.8 (-7.4, 8.4)	1.7 (-6.7, 9.5)	-1.7 (-15.5, 10.4)	2.3 (-16.1, 17.7)
		PAF[2]	1.1 (-6.8, 9.0)	1.9 (-5.9, 9.8)	-1.6 (-15.6, 12.4)	2.2 (-13.1, 17.5)
**Low education level**	Fixed effect model	RR	1.12 (1.02, 1.23)	1.09 (0.99, 1.20)	1.20 (1.06, 1.37)	Not applicable
		PAF[1]	4.6 (1.0, 8.2)	3.7 (-0.2, 7.4)	5.3 (0.8, 9.5)	Not applicable
		PAF[2]	3.0 (0.8, 5.2)	2.2 (-0.2, 4.6)	4.6 (1.6, 7.5)	Not applicable
	Random effect model	RR	1.17 (0.89, 1.53)	1.15 (0.86, 1.52)	1.03 (0.55, 1.92)	Not applicable
		PAF[1]	5.1 (-6.7, 15.6)	4.2 (-8.3, 15.2)	2.8 (-14.7, 17.6)	Not applicable
		PAF[2]	3.9 (-2.3, 10.2)	3.5 (-3.2, 10.1)	0.8 (-15.4, 17.1)	Not applicable
**Quit smoking**	Fixed effect model	RR	0.92 (0.81, 1.04)	0.91 (0.81, 1.04)	1.09 (0.90, 1.31)	1.01 (0.82, 1.23)
		PAF[1]	-0.8 (-2.2, 0.7)	-0.8 (-2.3, 0.6)	0.7 (-1.0, 2.5)	0.1 (-2.3, 2.4)
		PAF[2]	-1.2 (-3.1, 0.7)	-1.3 (-3.2, 0.6)	1.1 (-1.2, 3.4)	0.1 (-2.7, 2.8)
	Random effect model	RR	0.91 (0.63, 1.31)	0.92 (0.64, 1.32)	1.14 (0.63, 2.07)	0.96 (0.45, 2.03)
		PAF[1]	-1.2 (-5.3, 2.8)	-1.1 (-5.2, 2.8)	0.9 (-4.5, 6.0)	-0.6 (-11.0, 8.8)
		PAF[2]	-1.3 (-6.8, 4.1)	-1.2 (-6.7, 4.2)	1.7 (-5.5, 8.8)	-0.6 (-11.3, 10.1)
**Continued smoking**	Fixed effect model	RR	1.75 (1.50, 2.02)	1.69 (1.46, 1.97)	1.61 (1.19, 2.19)	1.30 (0.91, 1.86)
		PAF[1]	1.6 (0.6, 2.5)	1.5 (0.5, 2.4)	1.1 (0.0, 2.2)	0.7 (-0.3, 1.7)
		PAF[2]	2.2 (1.8, 2.6)	2.1 (1.7, 2.6)	2.0 (1.0, 2.9)	1.2 (-0.2, 2.6)
		RR	1.47 (0.92, 2.37)	1.47 (0.91, 2.36)	1.52 (0.71, 3.25)	1.13 (0.42, 3.05)
		PAF[1]	3.2 (-0.4, 6.6)	3.2 (-0.5, 6.7)	1.7 (-3.3, 6.5)	-0.0 (-5.2, 4.9)

	Random effect model	PAF[2]	1.7 (-0.0, 3.3)	1.7 (-0.0, 3.3)	1.8 (-0.8, 4.4)	0.6 (-4.0, 5.2)
**Quit alcohol consumption**	Fixed effect model	RR	1.00 (0.89, 1.12)	1.01 (0.90, 1.14)	1.01 (0.90, 1.14)	1.01 (0.88, 1.14)
		PAF[1]	-0.4 (-5.6, 4.5)	0.3 (-4.9, 5.3)	0.2 (-5.1, 5.1)	0.2 (-5.1, 5.3)
	Random effect model	RR	0.99 (0.69, 1.42)	1.02 (0.70, 1.47)	1.01 (0.70, 1.47)	0.99 (0.61, 1.61)
		PAF[1]	-0.4 (-22.2, 17.6)	1.1 (-21.2, 19.3)	1.0 (-21.2, 19.2)	-0.6 (-23.3, 18.0)
**Continued alcohol consumption**	Fixed effect model	RR	1.17 (1.01, 1.35)	1.13 (0.98, 1.31)	1.12 (0.89, 1.40)	1.12 (0.89, 1.41)
		PAF[1]	0.9 (-0.4, 2.2)	0.7 (-0.6, 2.0)	0.4 (-1.1, 1.9)	0.9 (-0.9, 2.7)
		PAF[2]	1.3 (0.2, 2.3)	1.0 (-0.1, 2.1)	0.9 (-0.8, 2.7)	1.0 (-0.8, 2.7)
	Random effect model	RR	1.10 (0.69, 1.77)	1.11 (0.69, 1.78)	1.20 (0.50, 2.89)	1.18 (0.51, 2.70)
		PAF[1]	0.6 (-3.2, 4.3)	0.4 (-3.4, 4.1)	-0.1 (-5.3, 4.9)	0.5 (-11.7, 11.4)
		PAF[2]	0.8 (-2.9, 4.6)	0.9 (-2.8, 4.6)	1.5 (-4.9, 7.8)	1.3 (-4.9, 7.5)

PAF, proportion attributable fraction; RR, risk ratio

Model 1, univariate

Model 2, adjusted for age, parity, maternal height, smoking, alcohol consumption, and pre-pregnancy underweight

Model 3, Model 2+gestational weight gain

Model 4, Model 2+education

Random effect model with pre-set heterogeneity at 80%

[1]PAF was calculated using PAF derived from each cohort.

[2]PAF was calculated using the pooled risk ratio estimated and the nationwide prevalence.

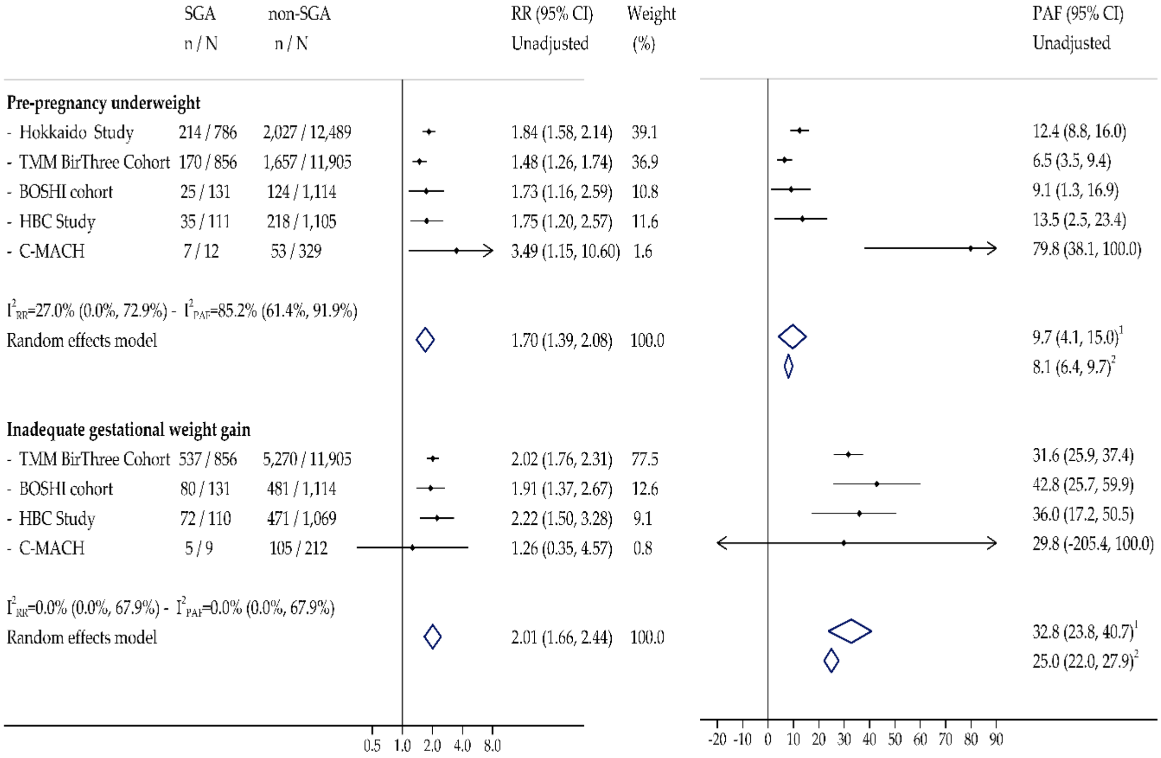

Figure S1 Results of meta-analyses of unadjusted risk ratio and population-attributable fraction of small-for-gestational age for pre-pregnancy underweight and inadequate gestational weight gain (Domestic criteria for small-for-gestational age).

Abbreviation: PAF, population attributable fraction; RR, risk ratio. I^2_{RR}, risk ratio heterogeneity, I^2_{PAF}, population attributable fraction heterogeneity.

[1]PAF was calculated using PAF derived from each cohort study.

[2]PAF was calculated using the pooled risk ratio estimated and the nationwide prevalence.

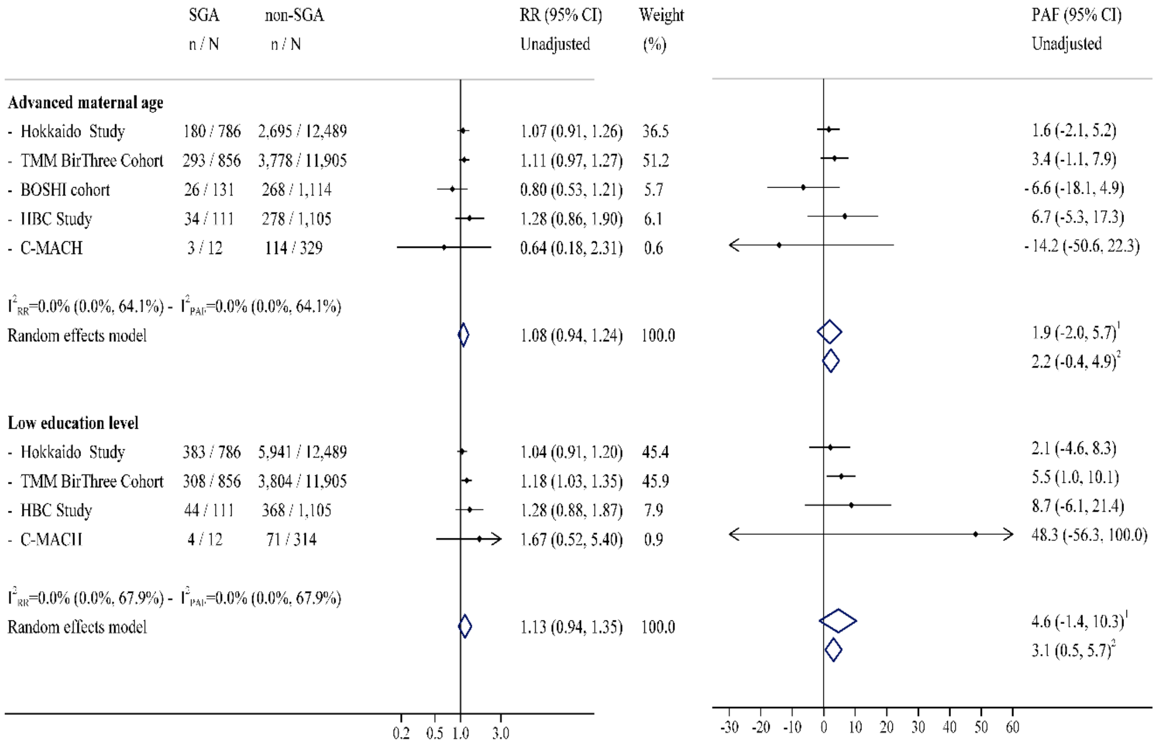

Figure S2 Results of meta-analyses of unadjusted risk ratio and population-attributable fraction of small-for-gestational age for advanced maternal age and low educational level (Domestic criteria for small-for-gestational age).

Abbreviation: PAF, population attributable fraction; RR, risk ratio. I^2_{RR}, risk ratio heterogeneity, I^2_{PAF}, population attributable fraction heterogeneity.

[1]PAF was calculated using PAF derived from each cohort study.

[2]PAF was calculated using the pooled risk ratio estimated and the nationwide prevalence.

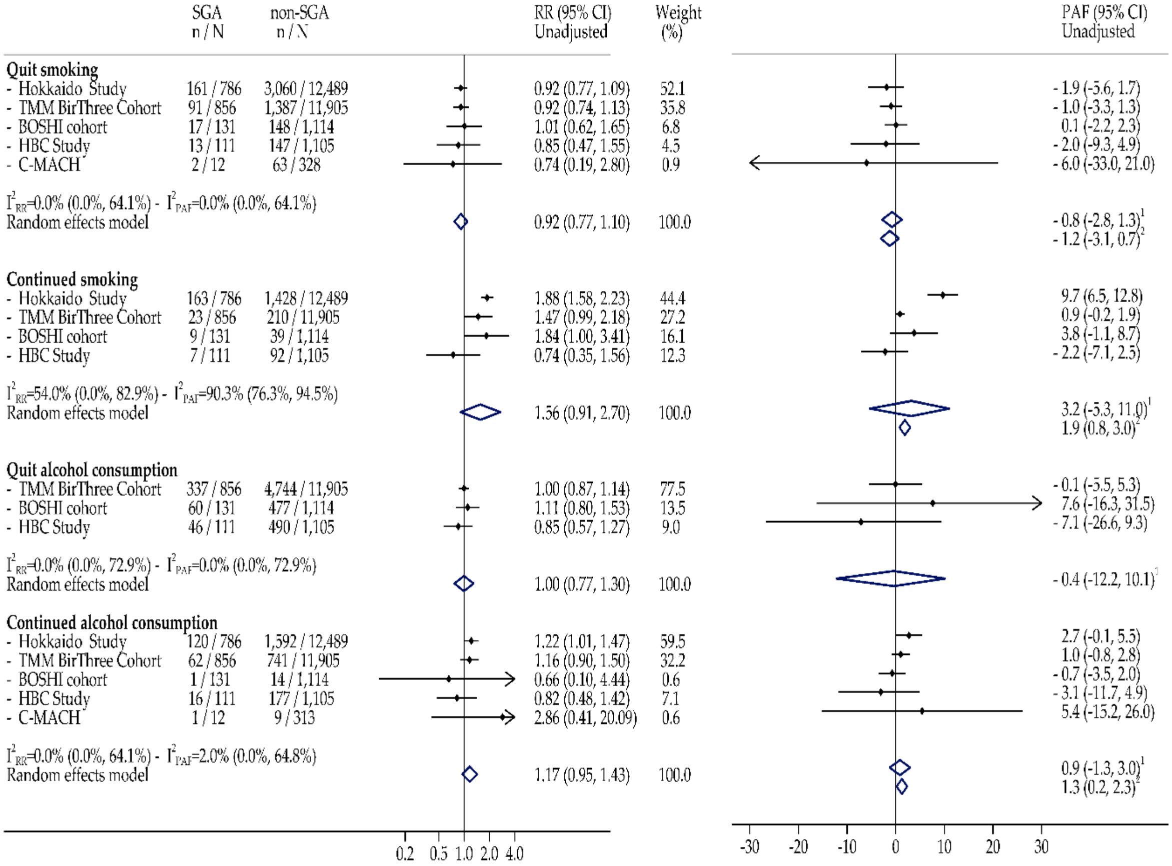

Figure S3 Results of meta-analyses of unadjusted risk ratio and population-attributable fraction of small-for-gestational age for smoking and alcohol consumption (Domestic criteria for small-for-gestational age). The reference groups of smoking status and alcohol consumption was never smoking and never alcohol consumption, respectively.

Abbreviation: PAF, population attributable fraction; RR, risk ratio. I^2_{RR}, risk ratio heterogeneity, I^2_{PAF}, population attributable fraction heterogeneity.

[1]PAF was calculated using PAF derived from each cohort study.

[2]PAF was calculated using the pooled risk ratio estimated and the nationwide prevalence.

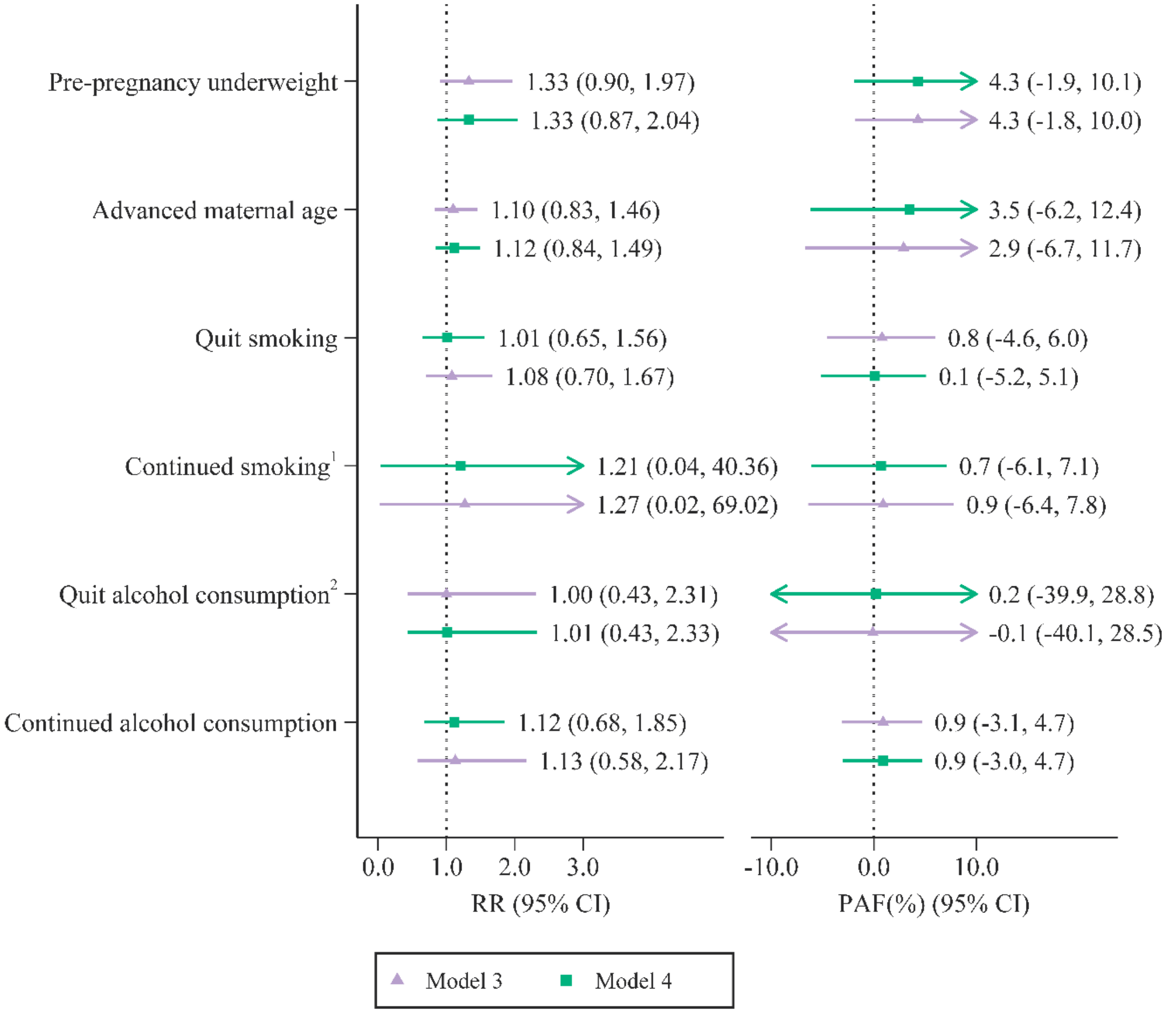

Figure S4 Results of the risk ratio and population-attributable fraction in models 3 and 4 (Domestic criteria for small-for-gestational age).

[1]C-MACH was not included in continued smoking because sample size of continuous smoking in SGA was zero.

[2]C-MACH was not included in quit alcohol consumption because they did not collect information.

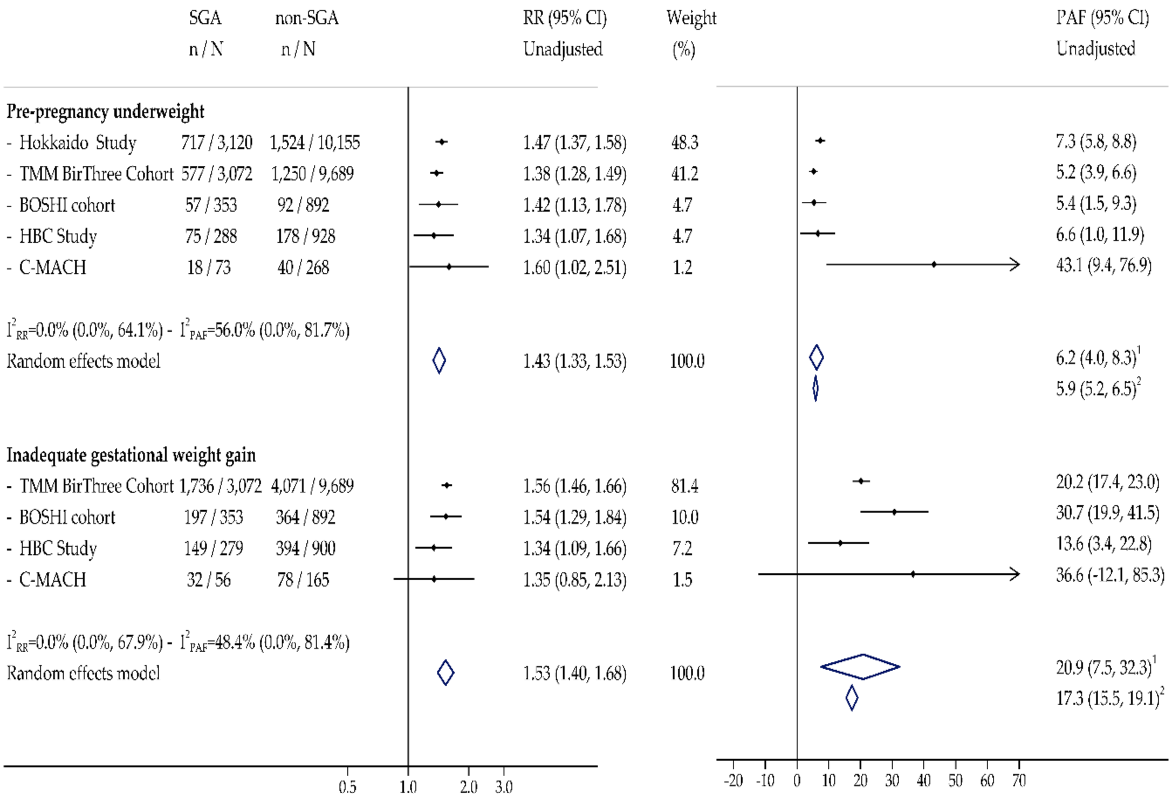

Figure S5 Results of the meta-analyses of the unadjusted risk ratio and population-attributable fraction of small-for-gestational age for pre-pregnancy underweight and inadequate gestational weight gain (International criteria for small-for-gestational age).

Abbreviation: PAF, population attributable fraction; RR, risk ratio. I^2_{RR}, risk ratio heterogeneity, I^2_{PAF}, population attributable fraction heterogeneity.

[1]PAF was calculated using PAF derived from each cohort study.

[2]PAF was calculated using the pooled risk ratio estimated and the nationwide prevalence.

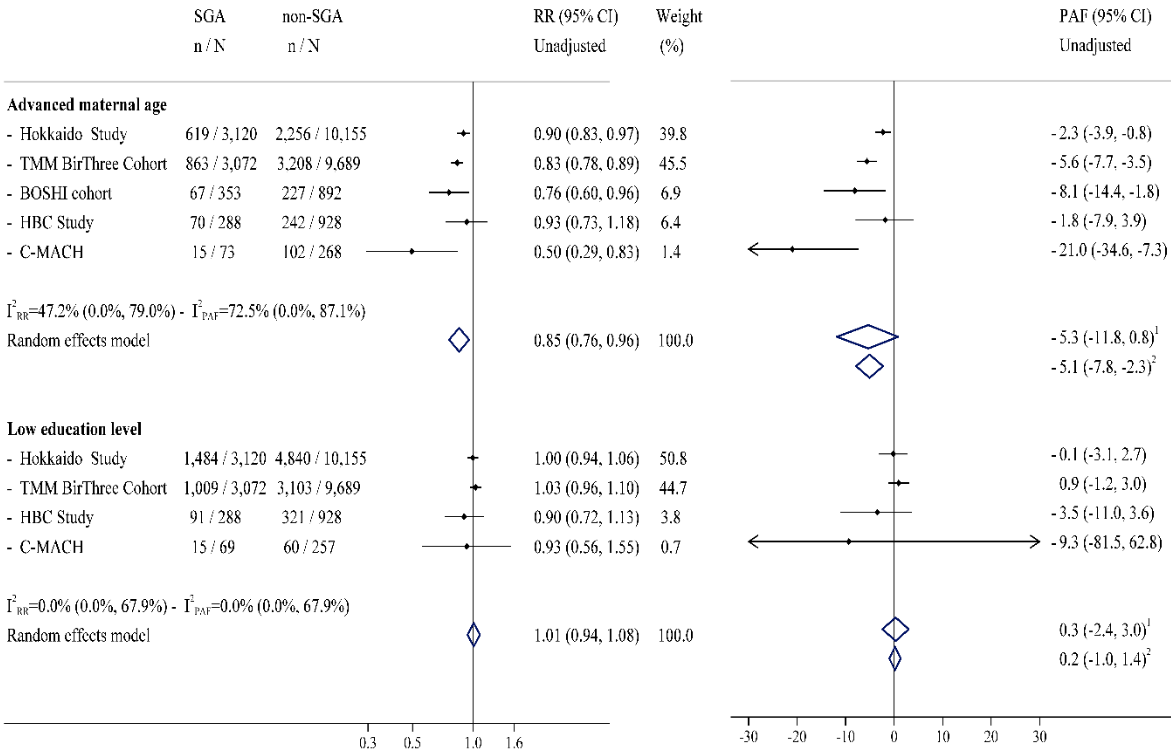

Figure S6 Results of the meta-analyses of the unadjusted risk ratio and population-attributable fraction of small-for-gestational age for advanced maternal age and low educational level (International criteria for small-for-gestational age).

Abbreviation: PAF, population attributable fraction; RR, risk ratio. I^2_{RR}, risk ratio heterogeneity, I^2_{PAF}, population attributable fraction heterogeneity.

[1]PAF was calculated using PAF derived from each cohort study.

[2]PAF was calculated using the pooled risk ratio estimated and the nationwide prevalence.

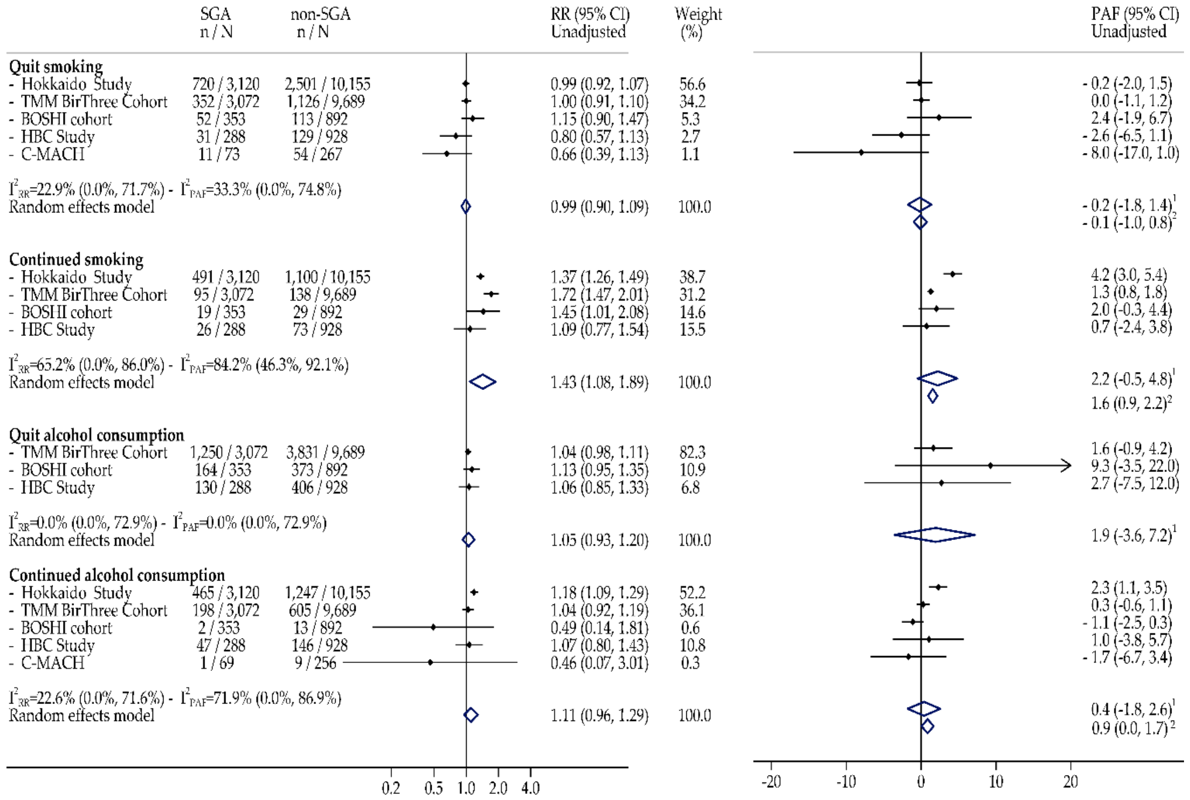

Figure S7 Results of meta-analyses of unadjusted risk ratio and population-attributable fraction of small-for-gestational age for smoking and alcohol consumption (International criteria for small-for-gestational age). The reference groups of smoking status and alcohol consumption was never smoking and never alcohol consumption, respectively.

Abbreviation: PAF, population attributable fraction; RR, risk ratio. I^2_{RR}, risk ratio heterogeneity, I^2_{PAF}, population attributable fraction heterogeneity.

[1]PAF was calculated using PAF derived from each cohort study.

[2]PAF was calculated using the pooled risk ratio estimated and the nationwide prevalence.

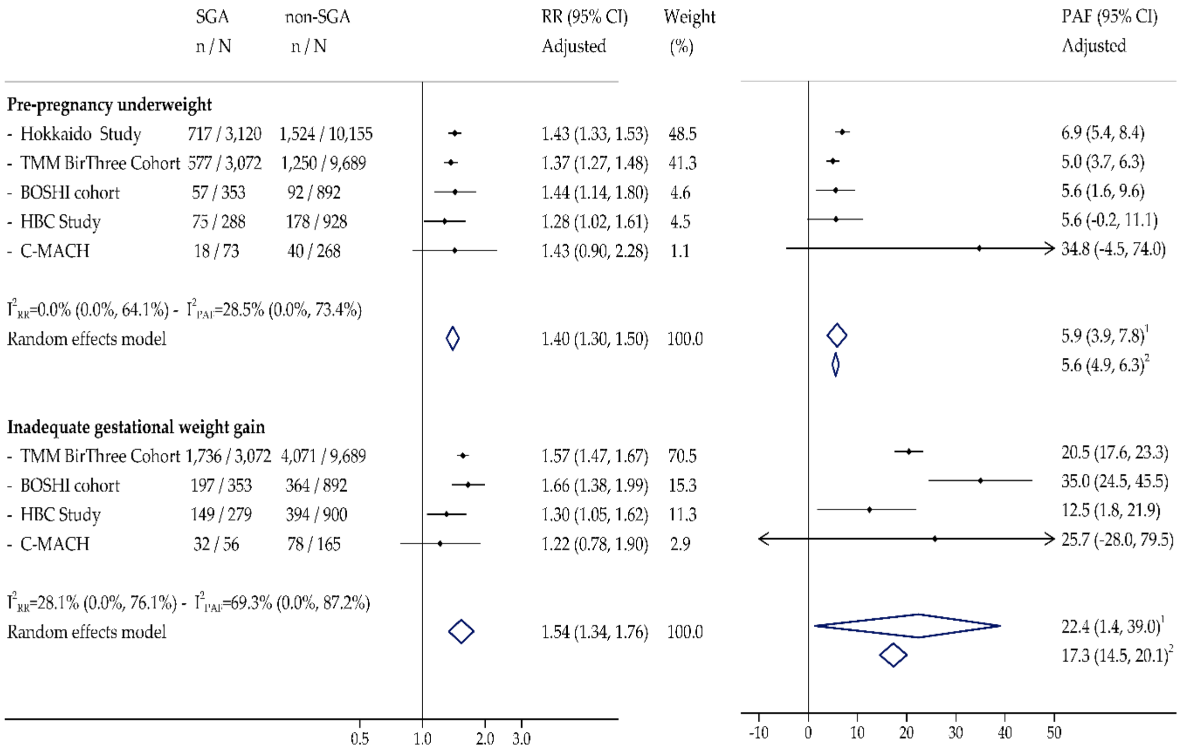

Figure S8 Results of the meta-analyses of the adjusted risk ratio and population-attributable fraction of small-for-gestational age for pre-pregnancy underweight and inadequate gestational weight gain (International criteria for small-for-gestational age).
Abbreviation: PAF, population attributable fraction; RR, risk ratio. I^2_{RR}, risk ratio heterogeneity, I^2_{PAF}, population attributable fraction heterogeneity.
[1]PAF was calculated using PAF derived from each cohort study.
[2]PAF was calculated using the pooled risk ratio estimated and the nationwide prevalence.

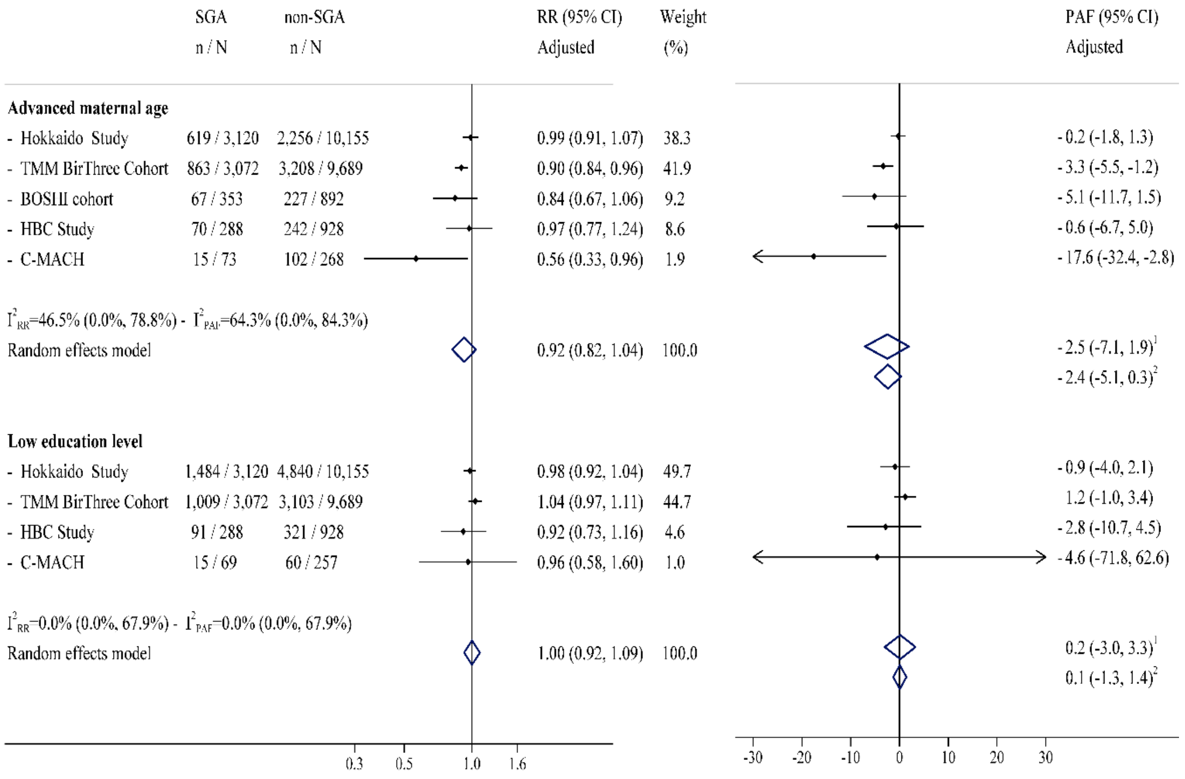

Figure S9 Results of the meta-analyses of the adjusted risk ratio and population-attributable fraction of small-for-gestational age for advanced maternal age, parity and low educational level (International criteria for SGA).

Abbreviation: PAF, population attributable fraction; RR, risk ratio. I^2_{RR}, risk ratio heterogeneity, I^2_{PAF}, population attributable fraction heterogeneity.

[1]PAF was calculated using PAF derived from each cohort study.

[2]PAF was calculated using the pooled risk ratio estimated and the nationwide prevalence.

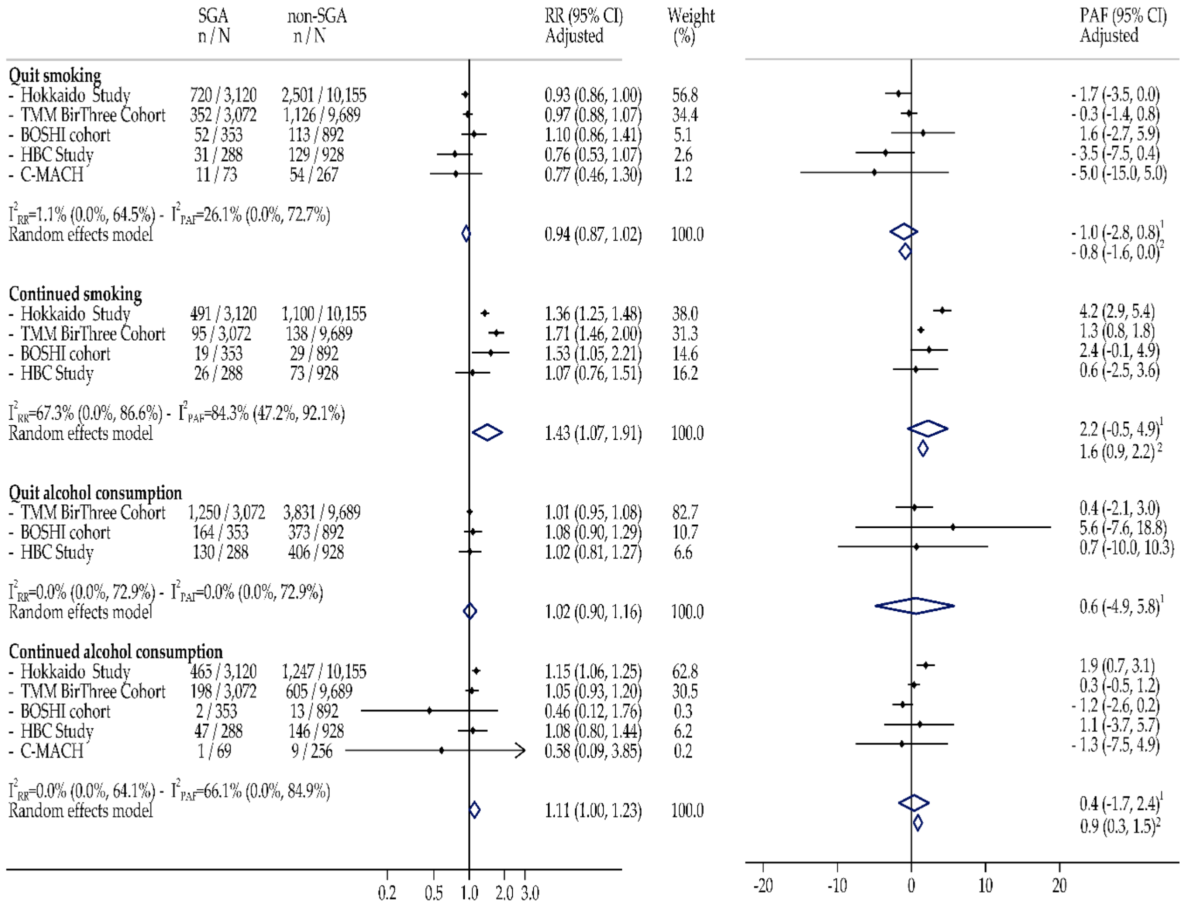

Figure S10 Results of meta-analyses of adjusted risk ratio and population-attributable fraction of small-for-gestational age for smoking and alcohol consumption (International criteria for small-for-gestational age). The reference groups of smoking status and alcohol consumption was never smoking and never alcohol consumption, respectively.

Abbreviation: PAF, population attributable fraction; RR, risk ratio. I^2_{RR}, risk ratio heterogeneity, I^2_{PAF}, population attributable fraction heterogeneity.

[1]PAF was calculated using PAF derived from each cohort study.

[2]PAF was calculated using the pooled risk ratio estimated and the nationwide prevalence.

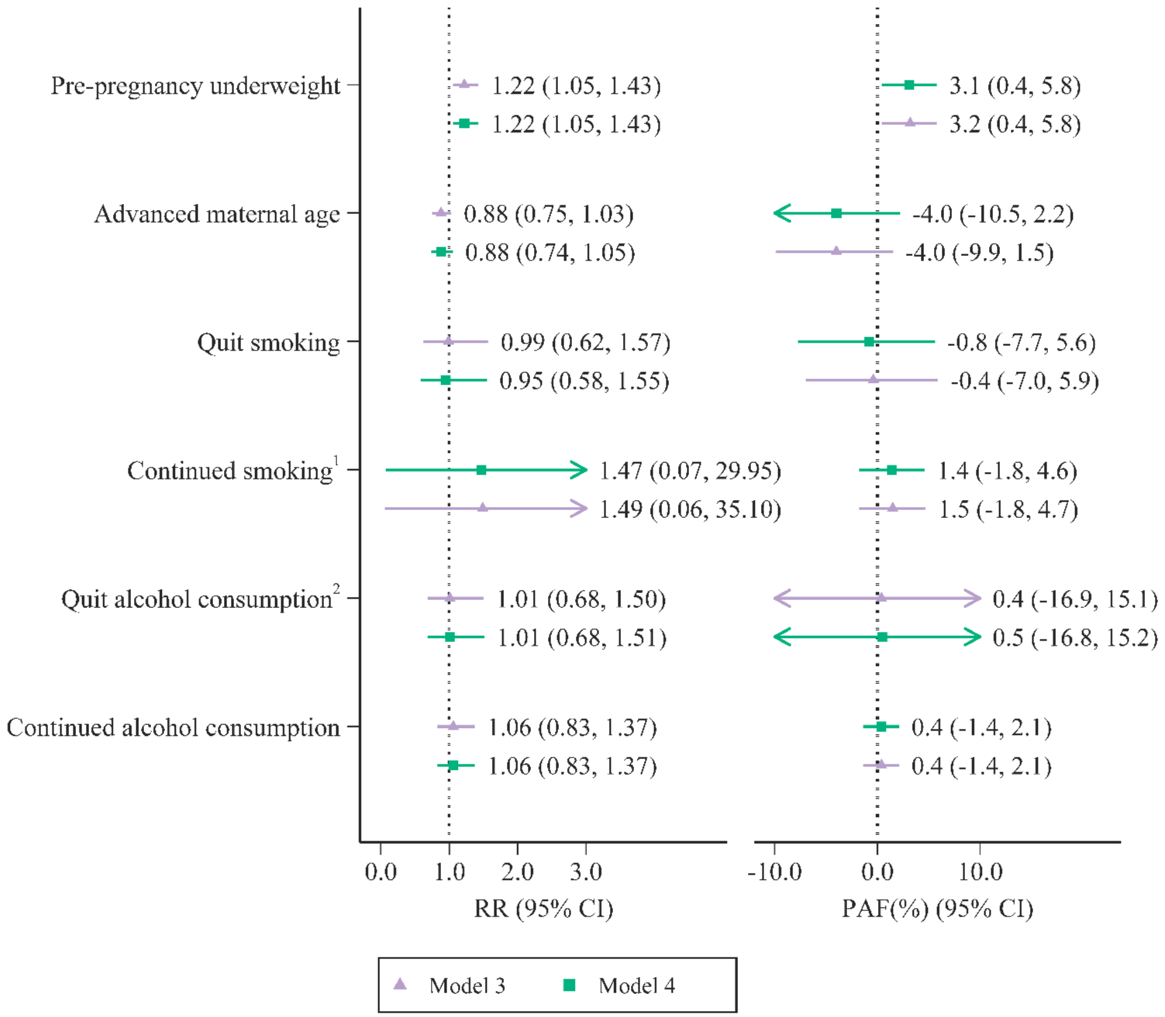

Figure S11 Results of the risk ratio and population-attributable fraction in models 3 and 4 (International criteria for small-for-gestational age).
[1]C-MACH was not included in continued smoking because sample size of continuous smoking in SGA was zero.
[2]C-MACH was not included in quit alcohol consumption because they did not collect information.
